# Feasibility of percutaneous coronary intervention before mitral NeoChord implantation: Single‐center early results

**DOI:** 10.1111/jocs.15953

**Published:** 2021-08-30

**Authors:** Nicola Pradegan, Augusto D'Onofrio, Lorenzo Longinotti, Giuseppe Evangelista, Florinda Mastro, Alessandro Fiocco, Matteo Nadali, Gino Gerosa

**Affiliations:** ^1^ Cardiac Surgery Unit, Department of Cardiac, Thoracic, Vascular Sciences and Public Health University of Padua Padua Italy

**Keywords:** coronary artery disease, micro‐invasive surgery, valve repair/replacement

## Abstract

**Background and Aim of the study:**

Micro‐invasive cardiac surgery identifies procedures performed off‐pump, on beating heart. Aim of this single‐center retrospective study was to assess early outcomes of a totally micro‐invasive strategy (percutaneous coronary intervention—PCI—followed by transapical off‐pump NeoChord mitral repair) in patients with concomitant coronary artery disease (CAD) and degenerative mitral regurgitation (MR).

**Methods:**

We analyzed early and 1‐year follow‐up data of patients who underwent a NeoChord procedure between November 2013 and May 2020, and preceded by PCI. Outcomes were defined according to Mitral Valve Academic Research Consortium (MVARC) definitions.

**Results:**

Among 220 patients who underwent NeoChord repair in the study period, 17 (7.7%) underwent PCI previously. CAD was an accidental finding during preoperative mitral evaluation in nine patients (52.9%; Group 1; with PCI occurring 2 months before NeoChord, interquartile range [IQR] = 1.0–2.7), while it was part of the past medical history in the remaining eight patients (47.1%; Group 2; with PCI occurring 30 months before NeoChord, IQR = 24.5–64.0). Twelve patients (70.6%) presented single‐vessel disease, two patients (11.8%) triple‐vessel disease. No surgical revisions for bleeding were required after NeoChord. At 1‐year follow‐up (*n* = 16), all patients were alive and did not experience major adverse events except for one reoperation due to late NeoChord failure. None required additional PCI.

**Conclusion:**

In our experience, PCI before NeoChord seems safe and effective, and performing PCI before NeoChord might not affect outcomes. A totally micro‐invasive strategy in selected patients suffering from MR and CAD should be considered as a reasonable alternative to conventional surgery.

## 1 INTRODUCTION

Recently, the new concept of micro‐invasive cardiac surgery has been introduced to identify procedures performed off‐pump, on beating heart (e.g., transcatheter aortic valve replacement, transcatheter mitral valve repair or replacement).^1^, ^2^

Mitral valve repair can be performed either with open‐heart procedures (full sternotomy or minimally invasive cardiac surgery) or through micro‐invasive transapical *neochordae* implantation. The former has shown well‐established early and long‐term results, while the latter has demonstrated promising early and 5‐year outcomes.^3^

Although open‐heart cardiac surgery remains the gold standard for patients with combined coronary artery disease (CAD) and mitral regurgitation (MR), a totally micro‐invasive strategy (percutaneous coronary intervention—PCI—+NeoChord) might allow to optimize outcomes especially in selected patients.

In the present single‐center, retrospective study, we aimed to analyze the early clinical outcomes of PCI followed by transapical off‐pump NeoChord mitral valve repair in patients with CAD and degenerative MR.

## 2 MATERIALS AND METHODS

Among all patients who underwent a NeoChord mitral repair procedure at the Padova University Hospital, we retrospectively analyzed early and 1‐year clinical and echocardiographic outcomes of those subjects who also underwent a previous PCI.

All enrolled patients had indications for surgical mitral repair due to degenerative MR according to current guidelines.^4^ Functional MR cases were excluded.

The choice to perform a NeoChord mitral repair was based on the following anatomical criteria: a mitral tissue overlap to obtain a potential postoperative coaptation length of 3–5 mm and the leaflet‐to‐annulus index (LAI) with a cutoff value of >1.25; the “surgically derived” morphological classification which includes four anatomical types (type A: isolated central posterior leaflet prolapse/flail; type B: posterior multisegment prolapse/flail; type C: anterior, bileaflet, or paracommissural disease; type D: leaflet and/or annular calcifications); cases showing mitral annular calcifications were excluded.^5^

All patients gave their informed consent for the procedure and for data collection for scientific purposes. Data collection of NeoChord procedures has been approved by the local Ethical Committee (No. AOP‐1772).

Outcome definitions were based on the Mitral Valve Academic Research Consortium (MVARC) guidelines.^6^ The primary endpoint was 1‐year MVARC patient success, and secondary endpoints were MVARC technical and procedural success (intraoperatively and at 30 days, respectively), and failure of the mitral repair (MR = severe). Clinical and echocardiographic follow‐up was performed for all patients at discharge and 1 year after NeoChord implantation. Postoperative MR was assessed with transthoracic echocardiograms according to the standard American Society of Echocardiography (ASE) modified criteria.^7^ MR was qualitatively defined by means of transthoracic echocardiography as trivial, mild, moderate, and severe.

### 2.1 Indications to PCI, PCI procedure, and NeoChord technique

PCI was performed according to the current guidelines.^8^, ^9^ Dual antiplatelet therapy (DAPT) had been started in each patient at the time of PCI, and continued for at least 6 months after the coronary procedure.^10^, ^11^ PCI was performed through a femoral or radial artery access.

**Figure 1:**
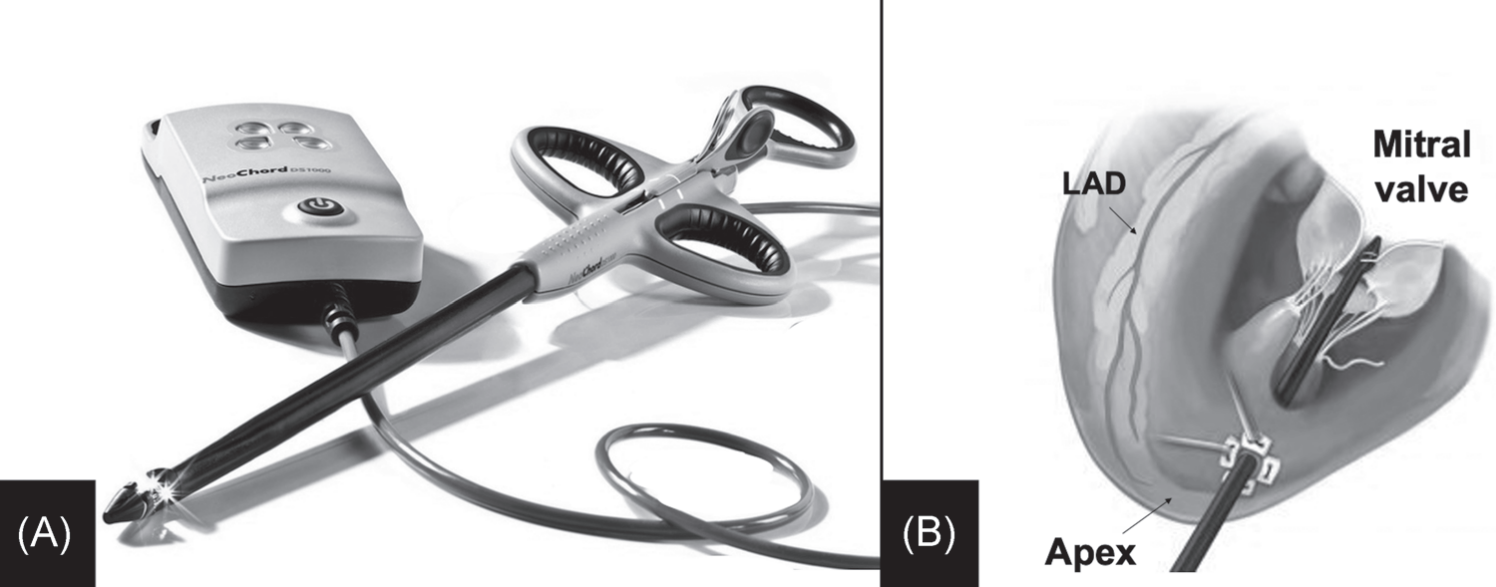
(A) Picture showing the NeoChord DS1000 device (NeoChord, Inc.). (B) Draw representing the device into the left ventricle and through the mitral valve, ready to release the *neochordae*. LAD, left anterior descending

The mitral NeoChord implantation is performed with the patient under general anesthesia, and access to the left heart is achieved through a left lateral mini‐thoracotomy in the fifth intercostal space. Two purse‐string sutures are placed 2–4 cm postero‐lateral from the apex of the left ventricle to pass in between the papillary muscles. After ventriculotomy, the NeoChord DS1000 device (NeoChord, Inc.; Figure 1A) is inserted in the left ventricle, and 2D‐ and 3D‐transesophageal echocardiographic imaging is used to guide the device to the prolapse/flail leaflet and implant the *neochordae* (Figure 1B). When the proper number of *neochordae* needed to correct MR has been implanted, they are tensioned under direct live‐3D transesophageal control. Finally, the tensioned *neochordae* are secured to the left ventricular epicardium using Teflon pledgets.^12^

### 2.2 Statistical analysis

Baseline and demographic categorical data were expressed as absolute numbers and percentages, while quantitative variables were expressed as medians (interquartile range [IQR]) or mean ± standard deviation as appropriate. Wilcoxon–Kruskal–Wallis test was performed for continuous variables and Pearson Chi‐square test for categorical ones. A *p* value of .05 was considered statistically significant. Computations have been performed using R 3.5.2 System and rms package.

## 3 RESULTS

Among 220 consecutive patients who underwent mitral repair with NeoChord implantation between November 2013 and May 2020, 17 patients (7.7%) were included in the analysis (mean age = 73 ± 9 years; *M* = 94%) and represent the population of our study. The median time between PCI and NeoChord repair was 4.9 months (IQR = 2.0–29.6). In nine patients (52.9%; Group 1), CAD was an incidental finding during the preoperative screening of the mitral valve disease, with a median time of 2 months between PCI and NeoChord repair (IQR = 1.0–2.7). Conversely, in eight patients (47.1%; Group 2), CAD was found as part of the past medical history, with PCI occurring 30 months before NeoChord repair (IQR = 24.5–64.0). In all these eight cases CAD was diagnosed in the context of an acute coronary syndrome. These patients underwent a re‐catheterization before the mitral procedure, showing patent stents and consequent no need for additional revascularization. Preoperative data have been summarized in Tables 1 and 2. Twelve patients (70.6%) presented with single‐vessel disease, 3 patients (17.6%) presented with two‐vessel disease, and 2 patients (11.8%) presented with triple‐vessel disease. CAD involved the left anterior descending artery in 10 patients (58.8%). Ten patients (58.8%) were on DAPT at the time of the mitral repair procedure (9 in Group 1, and 1 in Group 2). According to the anatomical classification,^4^ patients were distributed as follows: type A, eight patients (47.1%), type B, six patients (35.3%), and type C, three patients (17.6%). No type D patients were included.

**Table 1:** Preoperative demographic and clinical data

Variable	*n* (%), mean ± *SD*		
	Elective PCI during preoperative mitral workup (*n* = 9)	Past medical history of PCI (*n* = 8)	*p*
Age (years)	71 ± 9	75 ± 10	.48
Males	9 (100%)	7 (87.5%)	.27
Arterial hypertension	5 (55.6%)	7 (87.5%)	.15
Dyslipidemia	3 (33.3%)	8 (100%)	.004*
Diabetes	1 (11.1%)	1 (12.5%)	.93
H/o smoking	1 (11.1%)	2 (25.0%)	.45
Peripheral artery disease	1 (11.1%)	2 (25.0%)	.45
Cerebrovascular disease	0	0	
Preoperative serum creatinine (mg/dl)	1.0 ± 0.2	1.0 ± 0.3	1
Preoperative hemoglobin (g/dl)	14.0 ± 1.3	12.7 ± 1.9	.20
Preoperative BMI (kg/mq)	26 ± 1	26 ± 3	.89
Preoperative EF (%)	65 ± 6	58 ± 8	.11
Euroscore II (%)	1.3 ± 0.9	3.7 ± 2.8	.04*
STS score (%)	1.4 ± 1.4	3.9 ± 4.7	.07
Preoperative NYHA III–IV	3 (33.3%)	6 (75.0%)	.09
Preoperative CCS angina > 1	0	1 (12.5%)	.27

*Note:* Variables are expressed as number of patients, *n* and percentages, %, or mean ± *SD*.

Abbreviations: BMI, body mass index; CCS, Canadian Cardiovascular Society; EF ejection fraction; H/o, history of; NYHA, New York Heart Association; SD, standard deviation; STS, Society of Thoracic Surgery.

*Statistically significant *p* values.

**Table 2:** CAD involvement and PCI characteristics

Variable	*n* (%), mean ± *SD*, median (IQR)		
	Elective PCI during preoperative mitral workup (*n* = 9)	Past medical history of PCI (*n* = 8)	*p*
**Time between PCI and NeoChord repair (months)**	2.0 (1.0–2.7)	30.0 (24.5–64.0)	<.001*
**Preoperative CAD**			
One‐vessel	8 (88.9%)	4 (50.0%)	.08
Two‐vessels	1 (11.1%)	2 (25.0%)	.45
Three‐vessels	0	2 (25.0%)	.11
PCI ^(*n is referred to vessels, no patients)^			
Left anterior descending	4	6	.20
Circumflex artery	5	4	.82
Right coronary artery	1	4	.08
**Bare metal stent**	0	1	.39
**Drug‐eluting stent**	10	13	.39
**DAPT at the time of mitral repair**	9 (100%)	1 (12.5%)	<.001*

*Note:* Variables are expressed as number of patients, *n* and percentages, %, median and IQR, or mean ± *SD*.

Abbreviations: CAD, coronary artery disease; DAPT, dual antiplatelet therapy; IQR, interquartile range; PCI, percutaneous coronary intervention; SD, standard deviation.

*Statistically significant *p* values.

During the mitral procedure, the mean cell‐saved, processed blood volume for the study population was 499 ± 347 ml, with no differences between the two groups (*p* = .85). Five patients required blood transfusion with a maximum of two units transfused during the hospital stay. Remarkably, we did not encounter intraoperative complications related to DAPT and to CAD such as bleeding or intraoperative acute myocardial infarction. Only two patients (11.8%) presented minor bleeding according to the MVARC bleeding scale. Furthermore, we did not observe any major complications related to the preoperative PCI (acute myocardial infarction, cerebrovascular accident, vascular complications, and acute kidney injury). No patient required >24 h of invasive ventilation.

One high‐risk patient (EuroScore II = 8.7%) with severe right ventricular dysfunction, high pulmonary pressure, and COPD experienced sudden cardiac death during the hospital stay. The remaining 16 patients had an uneventful hospital stay and were discharged in good clinical conditions (Table 3).

**Table 3:** Intraoperative and postoperative outcomes

Variable	*n* (%), mean ± *SD*		
	Elective PCI during preoperative mitral workup (*n* = 9)	Past medical history of PCI (*n* = 8)	*p*
**NeoChord implanted on PML**	4 ± 1	3 ± 1	.17
**NeoChord implanted on AML**	3 neochordae in only 1 pt	1 ± 1	
**NeoChord procedural time (min)**	129 ± 30	116 ± 22	.61
**Cell‐saved blood volume (ml)**	483 ± 339	522 ± 389	.85
**RBC transfusions (U)**			
0	7	5	.49
1	0	2	.11
2	2	1	.60
**Mechanical ventilation time (h)**	3 ± 1	4 ± 7	.61
**MVARC technical success**	9 (100%)	8 (100%)	
**MVARC bleeding**			
No	0	0	
Minor	1 (12.5%)	1 (12.5%)	.93
Major	0	0	
Extensive	0	0	
**Life‐threatening or fatal**	0	0	
**MVARC vascular complications and access‐related complications**	0	0	
**MVARC neurological events**	0	0	
**MVARC acute myocardial infarction**	0	0	
**MVARC acute kidney injury**	1 (12.5%)	1 (12.5%)	.93
**New onset atrial fibrillation**	4 (44.4%)	3 (37.5%)	.77
**Deep wound infection**	0	0	
**ICU stay days > 1**	0	1 (12.5%)	.27
**Total hospital length of stay (days)**	7 ± 2	8 ± 2	.82
**Device related death**	0	0	
**Postoperative in‐hospital mortality**	0	1 (12.5%)	.27
**MVARC procedural success**	9 (100%)	7 (87.5%)	.27
**MR ≤ mild at discharge**	9 (100%)	7 (87.5%)	.27

*Note:* variables are expressed as number of patients, *n* and percentages, %, or mean ± *SD*.

Abbreviations: AML, anterior mitral leaflet; ICU, intensive care unit; MR, mitral regurgitation; MVARC, Mitral Valve Academic Research Consortium; PML, posterior mitral leaflet; RBC, red blood cells; SD, standard deviation.

At 1‐year follow‐up, all 16 patients were alive; of these, 15 were in good clinical status (NYHA Class I, CCS Class 1); one patient had severe MR due to recurrent prolapse after 2 months and underwent a successful transcatheter edge‐to‐edge repair. Thirteen patients (81.3%) presented with residual trivial/mild MR and 2 patients (12.5%) presented as asymptomatic with residual moderate MR. None of the patients presented acute coronary syndromes or ischemic symptoms, and none required coronary reintervention.

## 4 DISCUSSION

The main finding of the present study is that a total micro‐invasive strategy for selected patients with associated CAD and MR is safe and effective.

Furthermore, a history of PCI before NeoChord mitral repair, regardless of timing, does not affect post‐procedural outcomes. In fact, there are no differences in terms of postoperative morbidity and mortality as well as 1‐year follow‐up outcomes between the two groups.

According to STS adult cardiac surgery database, traditional surgical mitral repair shows 1.1% mortality, which increases to 6.2% when associated with CABG.^11^ For this reason, intraoperative and postoperative risks related to combined mitral and CAD surgery may be reduced by favoring lower‐risk procedures such as PCI and micro‐invasive mitral repair techniques in selected patients.

Among different mitral repair strategies for patients who present degenerative MR, the micro‐invasive off‐pump NeoChord mitral repair has shown to be a safe, and reproducible technique, with good outcomes at discharge, and clinical efficacy maintained up to 5 years of follow‐up.^12^, ^13^

In the setting of CAD and mitral valve disease, the less invasive strategy of PCI followed by minimally invasive valve surgery has also demonstrated positive early and midterm results.^14^, ^15^ However, these recent works have not considered micro‐invasive mitral procedures, which constitute a rapidly expanding field, and have the potential of being adopted as a valuable alternative to conventional or minimally invasive surgery in selected patients.^16^ Patient selection is crucial to understand who will benefit from these techniques. Regarding the NeoChord procedure, several echocardiographic parameters (LAI, morphological classification, and length of coaptation prediction index) have been introduced to help to standardize preoperative selection. The most recent evidence shows that NeoChord repair can be a reasonable alternative to conventional surgery for a subset of patients with MR in an early phase when the disease is limited to the leaflets and not extended to the annulus and/or to the left ventricle.^12^ In this study, the procedures were performed by the same operator (Gino Gerosa) and all cases were performed after the initial 40 cases (recognized by the CUSUM analysis as the threshold to standardize the procedure in all its technical aspects).^17^

In our cohort, a micro‐invasive treatment strategy resulted satisfactory in terms of reduced blood transfusions, reduced ventilation, and hospitalization times.

STS database demonstrates not only a higher mortality (6.2%) in patients undergoing MV repair and CABG, but also a significant higher rate of major bleedings (5.5%) and stroke (2.8%) than those observed in the present report.^18^

In patients with the previous PCI, undergoing minimally invasive mitral valve repair, Santana et al.^15^ showed promising outcomes in terms of postoperative cerebrovascular accident (1.1%), acute kidney injury (2.2%), and reoperation for bleeding (4.3%), with a low postoperative mortality (4.3%),^15^ similarly to our study. However, a fair comparison is not possible because of the limited number of patients treated in both series.

Albertini et al.^19^ have recently shown the feasibility of combining minimally invasive direct CABG (MIDCAB) with the NeoChord mitral repair procedure as a potential strategy to treat combined CAD and MV disease.

These two reports highlight the concept that the availability of innovative surgical procedures, along with PCI, makes this association a very attractive strategy, since it can combine the advantages of the two approaches.

This study carries important limitations; first, it was a retrospective analysis of a small study cohort. Besides, PCI procedures were performed at different centers, and it was not possible to perform a SYNTAX score analysis for all patients. Follow‐up testing for residual CAD disease such as stress test or coro‐CT scan was also lacking.

## 5 CONCLUSIONS

In conclusion, according to our data, PCI before NeoChord mitral repair procedures is a safe and effective strategy, and performing PCI before NeoChord does not affect outcomes. Therefore, a totally micro‐invasive strategy in selected patients suffering from MR and CAD should be considered as a reasonable alternative to conventional surgery.

## CONFLICT OF INTERESTS

Nicola Pradegan, Alessandro Fiocco, Matteo Nadali, and Gino Gerosa received travel grants from NeoChord Inc.

## AUTHOR CONTRIBUTIONS

*Nicola Pradegan*: data analysis/interpretation, drafting article; *Augusto D'Onofrio*: critical revision article; *Lorenzo Longinotti*: data analysis/interpretation; *Giuseppe Evangelista*: data analysis/interpretation; *Florinda Mastro*: data analysis/interpretation; *Alessandro Fiocco*: data analysis/interpretation; *Matteo Nadali*: data analysis/interpretation; *Gino Gerosa*: concept/design, critical revision of article.
